# Decoding drought resilience: a comprehensive exploration of the cotton Eceriferum (*CER*) gene family and its role in stress adaptation

**DOI:** 10.1186/s12870-024-05172-8

**Published:** 2024-05-29

**Authors:** Rasmieh Hamid, Zahra Ghorbanzadeh, Feba Jacob, Mojtaba Khayam Nekouei, Mehrshad Zeinalabedini, Mohsen Mardi, Akram Sadeghi, Mohammad Reza Ghaffari

**Affiliations:** 1 Department of Plant Breeding, Cotton Research Institute of Iran (CRII), Agricultural Research, Education and Extension Organization (AREEO), Gorgan, Iran; 2 Department of Systems Biology, Agricultural Biotechnology Research Institute of Iran (ABRII), Agricultural Research, Education and Extension Organization (AREEO), Karaj, Iran; 3 Centre for Plant Biotechnology and Molecular Biology, Kerala Agricultural University, Thrissur, India; 4 Faculty of Biological Science, University of Tarbiat Modares, Tehran, Iran; 5 Department of Microbial Biotechnology and Biosafety, Agricultural Biotechnology Research Institute of Iran (ABRII), Agricultural Research, Education and Extension Organization (AREEO), Karaj, Iran

**Keywords:** Eceriferum (*CER*), Biosynthesis of cuticle wax, micro-RNA, Gene ontology, Stress conditions, Synteny

## Abstract

**Background:**

The cuticular wax serves as a primary barrier that protects plants from environmental stresses. The Eceriferum (*CER*) gene family is associated with wax production and stress resistance.

**Results:**

In a genome-wide identification study, a total of 52 members of the *CER* family were discovered in four *Gossypium* species: G. *arboreum*, G. *barbadense*, G. *raimondii*, and G. *hirsutum*. There were variations in the physicochemical characteristics of the *Gossypium* CER *(*GCER*)* proteins. Evolutionary analysis classified the identified *GCERs* into five groups, with purifying selection emerging as the primary evolutionary force. Gene structure analysis revealed that the number of conserved motifs ranged from 1 to 15, and the number of exons varied from 3 to 13. Closely related *GCERs* exhibited similar conserved motifs and gene structures. Analyses of chromosomal positions, selection pressure, and collinearity revealed numerous fragment duplications in the *GCER* genes. Additionally, nine putative *ghr-miRNAs* targeting seven G. *hirsutum CER (GhCER)* genes were identified. Among them, three miRNAs, including *ghr-miR394*, *ghr-miR414d*, and *ghr-miR414f*, targeted *GhCER09A*, representing the most targeted gene. The prediction of transcription factors (TFs) and the visualization of the regulatory TF network revealed interactions with *GhCER* genes involving ERF, MYB, Dof, bHLH, and bZIP. Analysis of *cis*-regulatory elements suggests potential associations between the *CER* gene family of cotton and responses to abiotic stress, light, and other biological processes. Enrichment analysis demonstrated a robust correlation between *GhCER* genes and pathways associated with cutin biosynthesis, fatty acid biosynthesis, wax production, and stress response. Localization analysis showed that most GCER proteins are localized in the plasma membrane. Transcriptome and quantitative reverse transcription-polymerase chain reaction (qRT-PCR) expression assessments demonstrated that several *GhCER* genes, including *GhCER15D*, *GhCER04A*, *GhCER06A*, and *GhCER12D*, exhibited elevated expression levels in response to water deficiency stress compared to control conditions. The functional identification through virus-induced gene silencing (*VIGS*) highlighted the pivotal role of the *GhCER04A* gene in enhancing drought resistance by promoting increased tissue water retention.

**Conclusions:**

This investigation not only provides valuable evidence but also offers novel insights that contribute to a deeper understanding of the roles of *GhCER* genes in cotton, their role in adaptation to drought and other abiotic stress and their potential applications for cotton improvement.

**Supplementary Information:**

The online version contains supplementary material available at 10.1186/s12870-024-05172-8.

## Introduction

Environmental factors, such as biotic and abiotic stressors, significantly impact the development and growth of plants [1]. Plant cuticular wax plays a crucial role in the control of non-stomatal transpiration and defence against mechanical damage, pathogens, UV radiation and other abiotic and biotic stress factors [2]. The plant cuticle consists of a complex scaffold composed of a keratinous macromolecular structure and a series of organic, solvent-soluble lipids commonly referred to as waxes [3]. The process of wax biosynthesis comprises three main stages. First, C16 and C18 fatty acids are synthesised from plastids, which serve as the basic building blocks for all lipids. Subsequently, these fatty acids are elongated in the endoplasmic reticulum to form very long-chain fatty acids (VLCFAs) from C20 to C34. Finally, the VLCFAs are converted via the decarbonylation and acyl reduction pathway, leading to the formation of various waxy components such as alkanes, esters, triterpenes, primary and secondary alcohols, ketones and aldehydes [4, 5]. Wax-deficient plant leaves often have a higher transpiration rate, lower chlorophyll content and poor CO_2_ assimilation [6].

Eceriferum (*CER*) genes have been widely researched in numerous plant species [7–9] and play critical roles in *VLCFA* and wax production [10]. The first member, the *CER1* gene (*AtCER1*), encodes decarbonylase, an enzyme that plays a central role in wax biosynthesis and catalyses the conversion of long-chain aldehydes into alkanes [11]. This enzyme is mainly activated in response to biotic and abiotic stress factors [11]. *MdCER1-like* contributes significantly to wax metabolism and water storage in tissues and plays an important role in drought stress [12]. *OsCER1* has been shown to affect the production of VLCFAs and alkanes, as well as the differentiation of plastids and pollen formation in rice [13]. *AtCER2* is involved in both pollen envelope and cuticle formation [14], as well as in elongating *C28* [15]. In addition, overexpression of *BnCER1*-2 in oilseed rapeseed (*Brassica napus*) resulted in increased alkane production and improved drought tolerance [16]. *CER3* was linked to epidermal wax formation in *Arabidopsis* in response to moisture fluctuations [17]. The complex of *CER3* and *CER1* together catalyze the formation of alkanes from *VLCFA*-CoA [18]. Ectopic expression of *AtCER1* and *AtCER3* genes in tobacco enhanced cuticle wax production and mitigated water loss during drought stress [19]. *CER4* was found in various plant tissues like roots, stem, leaves, siliques and flowers [20].

Adenosine triphosphate binding cassette (ABC) proteins facilitate the transport of endogenous substrates across intracellular membranes as well as plasma. They transfer the epicuticular wax from the epidermal cells to the cuticle [21]. The *CER5* gene in *Arabidopsis* codes for an ABC transporter responsible for the export of wax to the epidermal plasma membrane [22]. Stem wax synthesis [11] and fatty acid production from C26 to C28 were increased by the *CER6* gene overexpression in *Arabidopsis* [23]. *Arabidopsis CER7* mutants showed downregulated expression of *CER3*/*WAX2* and promoted cellular events and thereby contributed to wax biosynthesis [24]. Another group of researchers found that in the biosynthetic pathway of cuticle wax, there exists a functional overlap between the mutant of *CER8/LACS* (long-chain acyl-CoA synthetase-1) in *Arabidopsis* and *LACS2* [25]. The *CER9*, in *Arabidopsis*, codes for a putative E3 ubiquitin ligase associated with cuticle biosynthesis. It also maintains the water status in plant by contributing to the abscisic acid (ABA) signalling in seed development and germination [9]. *CER10* was also found to be associated with VLCFA biosynthesis [26], and further studies showed that the mutants of *CER10* exhibited increased non-stomatal water loss and enhanced resilience to drought [22]. The mutants overexpressing *CER26* showed a glossy stem phenotype. *CER60* was associated with VLCFA, elongation of C28 to C30 activity, and production of trace amounts of VLCFA after expression in yeast [27].

In delving deeper into the intricacies of *CER* gene families across various plant species, researchers systematically examined these families in jujube, apple, sunflower, maracuja, and *Castanea mollissima*, identifying 29, 10, 37, and 29 *CER* gene family members, respectively [28–32]. However, the knowledge about the *CER* family in cotton (*Gossypium* sp.), the world’s largest fibre crop [33], is less extensive. Due to the complex origin of the allotetraploid species, housing two genomes (A and D), systematic studies of cotton gene families have progressed slowly. Given the economic and social importance of cotton, coupled with the critical roles of *CER* in plants, investigations into the cotton *CER* gene family are imperative for understanding the biological processes underpinning stress responses and development in cotton.

This study identified the *CER* gene family from G. *arboreum*, G. *raimondii*, G. *barbadense*, and G. *hirsutum*. Various characteristics, including gene structure, conserved motifs and domains, subcellular localization, synteny relationships, chromosome localization, evolution and the gene expression patterns were systematically characterized and analyzed. The location of *cis*-acting elements in *CER* promoters was also studied, providing crucial insights into the function of *GCER*s. Furthermore, we delved into the expression of *GhCER04A* and the gene’s functions using the virus-induced gene silencing (*VIGS*) procedure to elucidate its functions in cotton during drought stress. Our findings lay the groundwork for comprehending the mechanisms regulating cotton *CER*s during drought stress, ultimately bolstering our ability to enhance genetic diversity for stress resistance.

## Results

### Identification of genes encoding *CER* across the entire genome in diploid and tetraploid cotton

A total of 9, 10, 17 and 16 CER proteins were identified in the genomes of G. *arboreum*, G. *raimondii*, G. *hirsutum* and G. *barbadense* respectively. Each *CER* gene was labeled on the basis of its location on the chromosome. The length of the amino acid residues of these cotton CER proteins ranged from 117 to 857 in G. *arboreum*, 617 to 671 in G. *raimondii*, 329 to 627 in G. *barbadense* and in G. *hirsutum*. The isoelectric points varied from 7.389 to 9.166 in G. *arboreum*, from 7.717 to 9.045 in G. *raimondii*, from 6.988 to 9.404 in G. *barbadense* and from 7.083 to 9.591 in G. *hirsutum*. Accordingly, the molecular weights ranged from 12.994 to 97.254 kDa in G. *arboreum*, 71.362 to 77.861 kDa in G. *raimondii*, 38.906 to 72.537 kDa in G. *barbadense* and 38.82 to 72.567 kDa in G. *hirsutum*. The WoLF PSORT website was used for predicting CER proteins subcellular localization, and the anticipated outcomes are illustrated in Supplementary Fig. 1a-d. The prediction of the localization of majority of the cotton CER proteins was in the plasma membrane, while only a few were localized in the cytoplasm, chloroplast, endoplasmic reticulum (E.R.), and mitochondria. Specifics about the nucleic acid and protein sequences of all 52 *CERs* obtained from all four cotton species can be found in Supplementary Table S1.

### Cotton CER protein’s phylogenetic classification

**Fig. 1:**
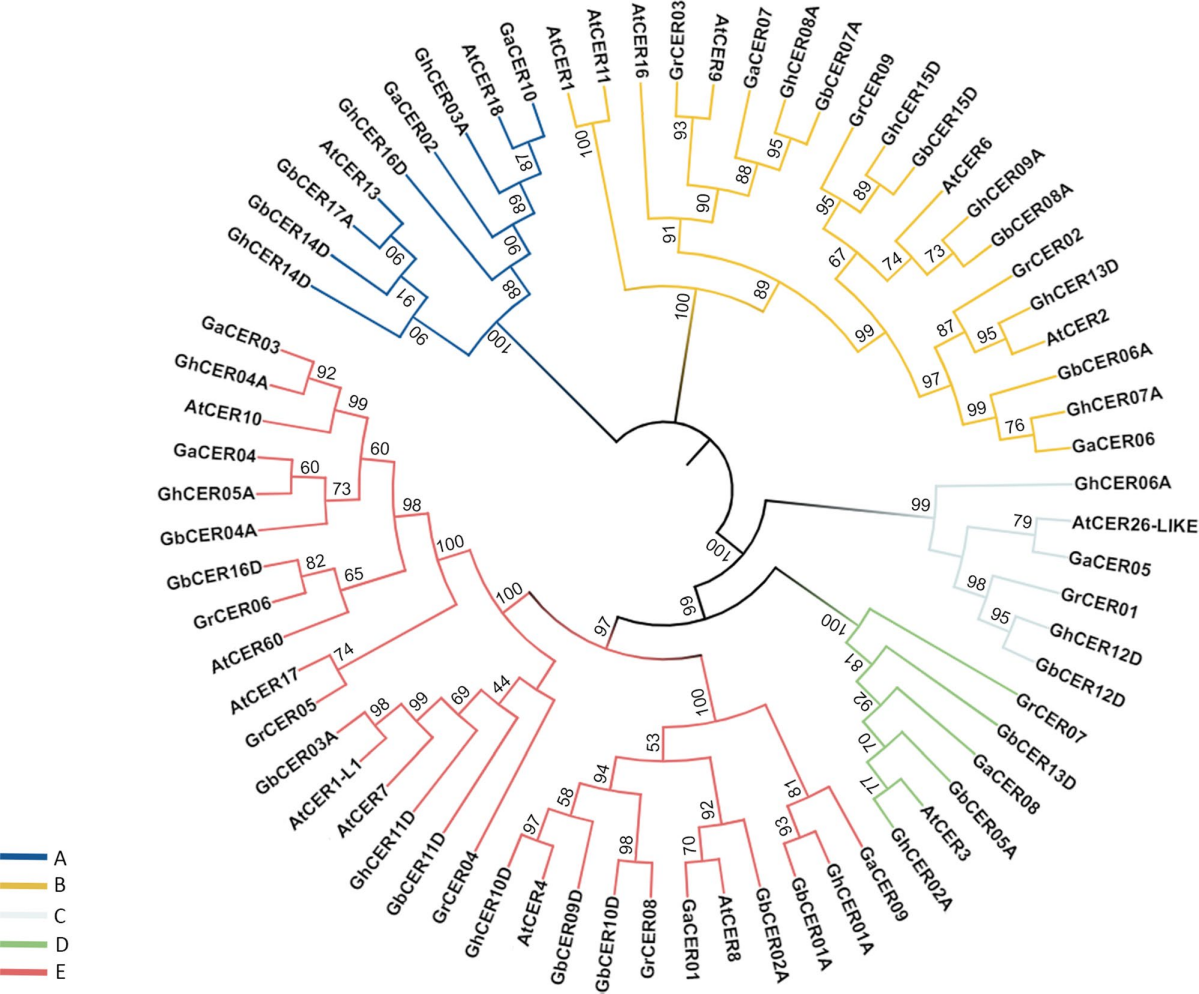
The phylogenetic analysis of *CER* genes among Arabidopsis and four cotton species. The phylogenetic tree was constructed using the program MEGA11.0. (The likelihood) algorithm, bootstrap value = 1000). The prefixes Ga, Gr, Gh and Gb represent G. *arboretum*, G. *raimondii*, G. *hirsutum* and G. *barbadense*, respectively

The phylogenetic tree constructed for *Arabidopsis thaliana* CER protein sequences and four cotton species classified all cotton *CERs* into five lineages (A, B, C, D, and E) (Fig. 1). The distribution of *CER* genes among various lineages displayed variation, with the greatest count of genes (28 genes) in lineage E and the smallest count of genes (six genes) in lineage C. The alignment and evolution results show that the CER proteins of the allotetraploid *Gossypium* species have greater homology with their own diploid ancestors than the CER proteins of *A. thaliana*. Moreover, in the majority of branches within the phylogenetic tree, genes originating from the A genome or the D genome tended to cluster together, except for a specific branch (D) where exclusively genes from the A subgenome were observed. Additionally, in most clusters, genes from both tetraploid and diploid species were grouped together. This aligns with the prior discovery indicating that the A genome and D genome of tetraploid cotton originated through the hybridization and duplication of two diploid cotton species [34, 35].

### Chromosomal distribution, multiple synteny, gene duplication, and collinearity analysis of *CER* genes

**Fig. 2:**
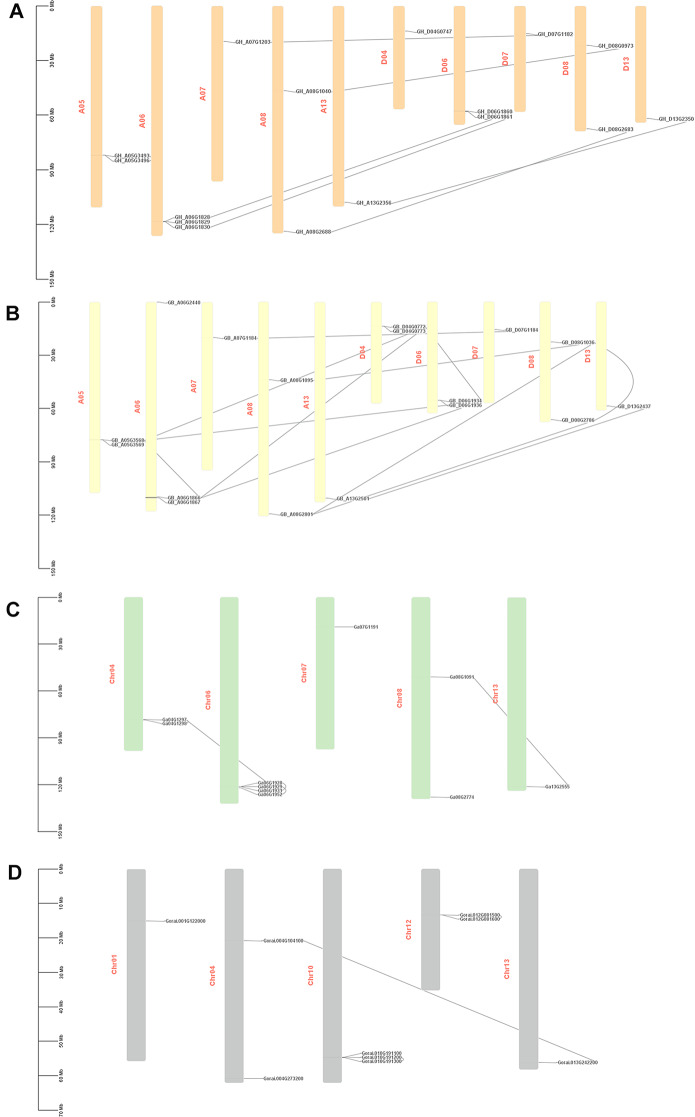
Chromosomal locations and gene duplication events of *CER* genes. The corresponding chromosome numbers are given to the left of each bar. *CER* gene pairs resulting from segmental and tandem duplications are connected by lines. Distribution of *CER* genes on chromosomes of G. *hirsutum* (**A**), G. *barbadense* (**B**), G. *arboreum* (**C**), G. *raimondii* (**D**)

To examine the chromosomal arrangement, the genomic positions of *CERs* were charted using the genome annotation files of four species. Sixteen *GhCERs* were haphazardly distributed on 10 chromosomes, with 2, 3, 1, 2, and 1 found on chromosomes A05, A06, A07, A08, and A13, respectively, while 1, 2, 1, 2, and 1 were found on chromosomes D04, D06, D07, D08, and D13, respectively (Fig. 2a). Seventeen *GbCERs* were unevenly distributed across 10 chromosomes and one scaffold (Fig. 2b). Unlike *GhCERs*, the arrangement of *GbCERs* across chromosomes At01-13 and Dt01-13 exhibited a comparable pattern in terms of both quantity and consistency. 10 *GaCERs* and 9 *GrCERs* were unevenly distributed among six chromosomes (Fig. 2c and d). In general, all *GhCERs*, *GbCERs* and *GaCERs* were mainly distributed on chromosomes 5, 7, 8, 9, 11, 12 and 13, but less so on 2 and 10. Improbably, most *GrCERs* were distributed on chromosomes 10 and 12 and less so on chromosomes 2, 3, 5 and 11.

To explore the evolutionary implications of polyploidization and hybridization, we studied the duplication patterns of *CER* genes in the four species of cotton. The *CER* genes of *G. arboreum*, *G. hirsutum*, G. *barbadense*, and *G. raimondii* were shown to have segmental or whole genome duplication (WGD). Nevertheless, each species exhibited three tandem duplications, and the dispersed gene duplication type was observed in four *CER* genes for *G. arboreum* and three for G. *raimondii*. Additionally, one *CER* gene from G. *hirsutum* and G. *arboreum* each, showed proximal gene duplications (Table S2). In brief, the *CER* gene family expansion in cotton is predominantly ascribed to either segmental duplications or whole-genome duplications.

**Fig. 3:**
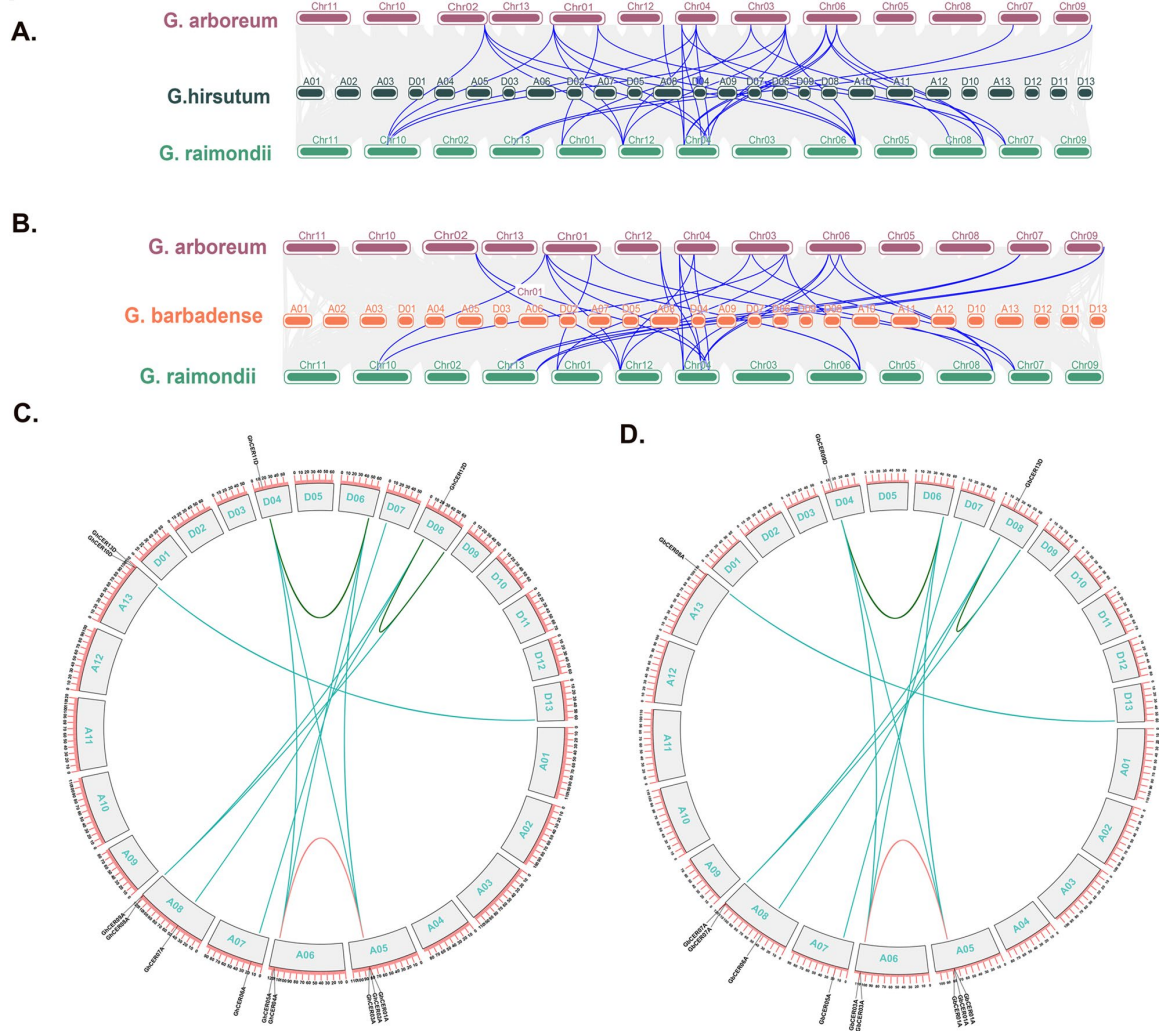
Multiple synteny analysis of cotton *CER* genes in cotton. Multiple synteny analysis was used to reveal the orthologous relationship of *CER* genes between **(A)** G. *hirsutum* and its ancestors G. *arboreum*, G. *raimondii* and **(B)** G. *barbadense* and its ancestors G. *arboreum*, G. *raimondii*. The chromosomes of the different cotton species are represented by different colours. **(C)** Collinearity analysis of the *CER* genes of G. *hirsutum*. and **(D)** collinearity analysis of the *CER* genes of G. *barbadense*. A01 to A13 represent chromosomes of the A subgenome, while D01 to D13 represent chromosomes of the D *subgenome*

The analysis of multiple synteny between the *CER* genes across G. *arboreum*, G. *raimondii*, G. *hirsutum* and G. *barbadense* unveiled 64 orthologous/paralogous gene pairs. These encompass seven gene pairs within G. *hirsutum*, 13 pairs between G. *barbadense* and G. *hirsutum*, six between G. *hirsutum* and G. *arboreum*, six between G. *raimondii* and G. *hirsutum*, 12 within G. *barbadense*, five between G. *barbadense* and G. *arboreum*, six between G. *raimondii* and G. *barbadense*, and one between G. *raimondii* and G. *arboreum* (Fig. 3a; b Table S3). We used a collinearity analysis to look at the positional relationships between the A and D subgenomes of *G. hirsutum* and *G. barbadense* (Fig. 3c, d). In *G*. *hirsutum*, there were a total of 13 pairs of orthologous or paralogous genes. Likewise, G. *barbadense* exhibited a total of 13 pairs of orthologous or paralogous genes.

Moreover, we utilized the ratio of non-synonymous substitutions (Ka) to synonymous substitutions (Ks) to assess the type of selective pressure acting on 64 duplicated *CER* gene pairs derived from eight combinations of four cotton species (G. *hirsutum* vs. G. *hirsutum*, G. *hirsutum* vs. G. *arboreum*, G. *hirsutum* vs. G. *barbadense*, G. *hirsutum* vs. G. *raimondii*., G. *barbadense* vs. G. *barbadense*, G. *barbadense* vs. G. *arboreum*, G. *barbadense* vs. G. *raimondii*, G. *raimondii* vs. G. *arboreum*) (Table S3). The results indicated that the Ka/Ks ratios for three gene pairs (*GB_D08G1036*/*GH_A08G1040*, *GB*_*A13G2501*/*GH_A13G2356*, and *GB_A07G1184*/*GH_A07G1203*) exceeded 1, signifying accelerated evolution in these pairs. In total, 50 gene pairs exhibited a Ka/Ks ratio below 0.5, while 11 gene pairs displayed a ratio ranging between 0.5 and 1. This observation leads us to suggest that these gene pairs are under robust purifying selection.

### Structural analysis of the cotton *CER* genes

**Fig. 4:**
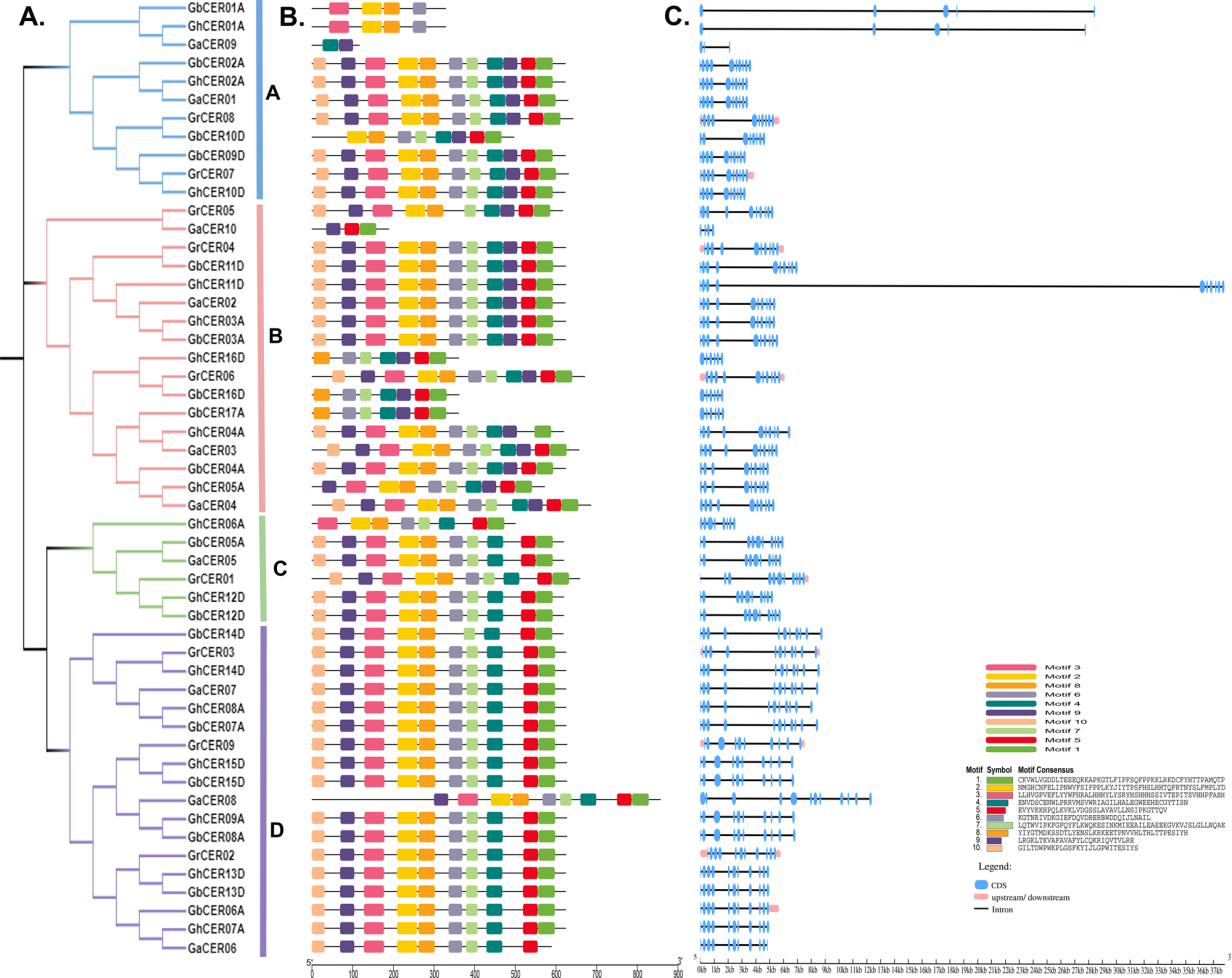
Phylogenetic relationships and structural characteristics of *CER* genes. **(A)** Phylogenetic analysis using the likelihood method for the *CER* family in four cotton species. **(B)** Patterns of predicted motifs within CER proteins. **(C)** Gene structures of the *CER* genes. Pink symbols represent untranslated 5′ and 3′ regions, blue symbols represent coding sequences (CDS), and lines indicate introns

Variations in gene structure are crucial in the evolutionary dynamics of a gene family. To enhance our comprehension of the *CER* genes’ structure in cotton, we scrutinized the structure of sequence, motifs, and conserved domains of these genes through analysis utilizing the phylogenetic tree (Fig. 4a). Using MEME, ten motifs were predicted in the CER proteins (labelled motifs 1 to 10). Shared motifs and comparable motif arrangements were identified in the majority of CER proteins within a given subfamily, implying a conserved structure among these proteins. With the exception of *GaCER09* and *GaCER10*, more than ten motifs were found in subfamily I and II proteins. Motifs 3 and 3 were absent in *GhCER06A* and *GrCER01* in subfamily III (Fig. 4b). Whether these specific patterns influence the function of the CER proteins is still under investigation. With the exception of *GaCER08* and *GaCER06*, all proteins of subgroup IV show the same pattern and the same number of motifs. In addition, the proteins of subfamily III showed the greatest variation in pattern and number of motifs.

The *CER* genes’ intron/exon structure was then analyzed. The structural similarities were evident among *CER* genes within the same subgroup, particularly noticeable in subfamily IV, which exhibited 11 exons. This suggests that the structures of *CER* genes are conserved across the four species. Nevertheless, within subclade I, one gene (*GaCER*09) featured 3 exons, *Gh/GbCER*01 had 5 exons, while all remaining genes exhibited 10–12 exons. In subclade II, one *CER* gene in cotton (*GaCER*10) had 4 exons, and three *CER* genes (*GbCER*16D, *GbCER*17A and *GhCER*16D) had 6 exons (Fig. 4c). These findings suggest that genes with varying number of exon/introns may serve distinct biological functions.

### Examination of the *cis*-acting elements in cotton species’ *CER* genes

**Fig. 5:**
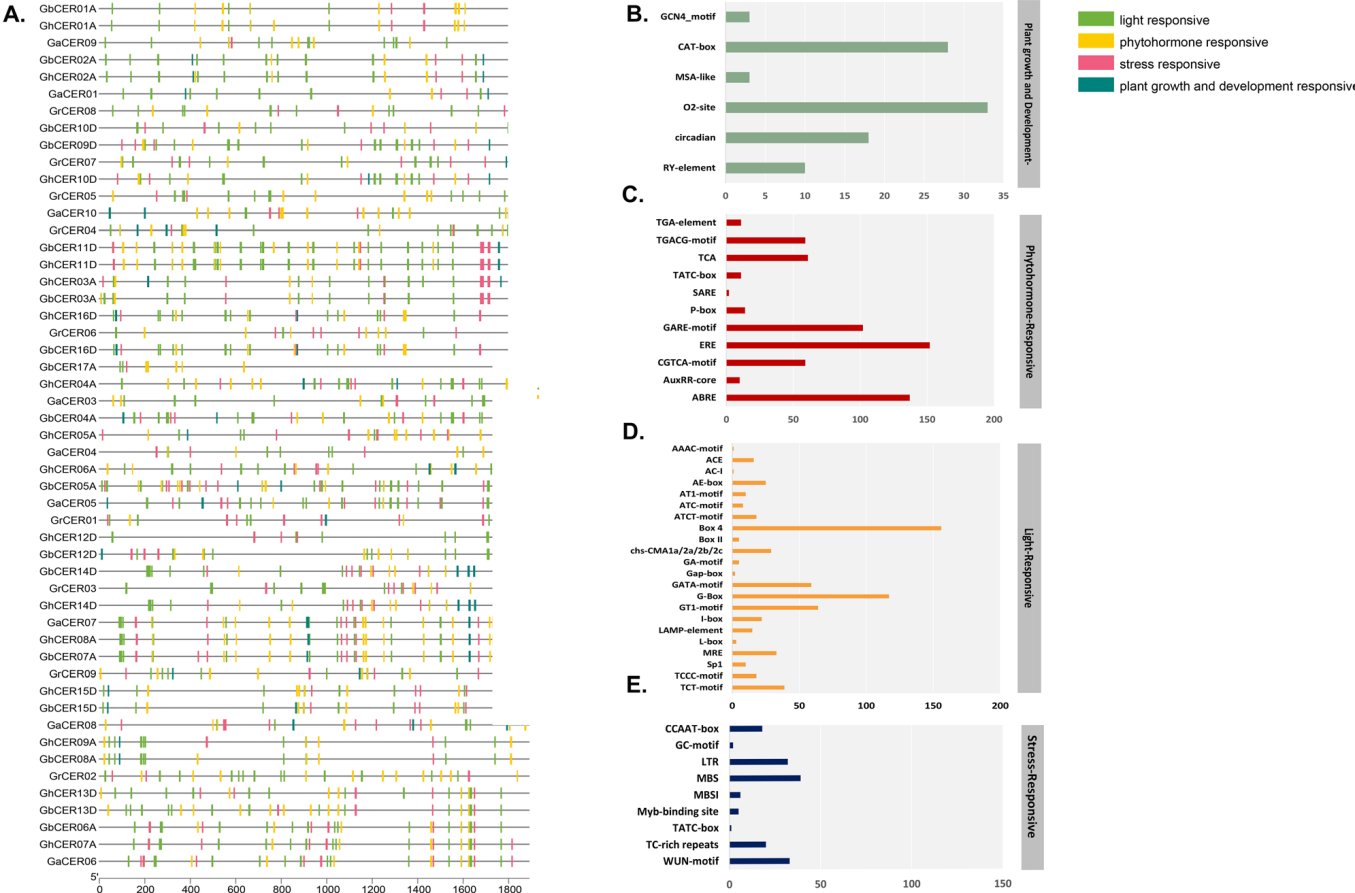
**A.** Analysis of cis-regulatory elements on CER genes. **B**. Total number of cis-elements and CER genes involved in four categories. Percentage (%) ratio of numerous cis-elements from each category is shown in bar graphs; **B.** plant growth and development responsive, **(C)** phytohormone responsive, **(D)** light responsive, **(E)** stress responsive

The gene expression regulation is orchestrated through upstream promoters housing numerous *cis*-acting elements. In order to obtain a better knowledge of the regulatory mechanisms and possible functions of the target genes we identified the *cis*-acting elements by scrutinizing the 2000 base pairs upstream of each *CER* gene’s predicted translation start site. The *CER* gene promoters primarily featured four categories of cis-regulatory elements: those responding to light, phytohormones, stress and growth and development (Fig. 5a, Table S4). Among these classifications, the light-responsive elements demonstrated the greatest prevalence of *cis*-elements, with the phytohormone-responsive and stress-responsive categories following in sequence. Conversely, the category linked to plant growth and development exhibited the lowest count of *cis*-elements.

Within the category of plant growth and development, the predominant *cis*-element was the O_2_ site at 34%, followed by the CAT box at 29%, circadian elements at 18% and the lowest proportion, 3%, attributed to the GCN4 motif. Moreover, the category related to plant growth and development was further subdivided into four subcategories, specifically metabolism, the cell cycle, meristem, and the circadian system. Metabolism-related *cis*-elements included the O_2_ site, while cell cycle-related elements included MSA-like elements. Meristem-associated *cis*-elements included the GCN4 motifs and CAT box, which are known to be associated with meristem expression, and circadian-associated *cis*-elements included Circadian and RY elements (Fig. 5b). The phytohormone-responsive category encompassed various *cis*-elements, including ABRE (abscisic acid responsive), ERE (ethylene responsive), AuxRR core (auxin responsive), TATC box (gibberellin responsive), P-box, SARE (salicylic acid responsive), GARE motif, TGACG motif (methyl jasmonate responsive), TCA element, and TGA element. Notably, ERE held the highest proportion at 29%. Following closely were ABRE at 25%, and TCA and CGTCA motifs at 11% each. In contrast, AuxRR-core and SARE exhibited the lowest representation, with each constituting only 1% of *cis*-elements (Fig. 5c). Within the light-responsive category, various *cis*-elements were identified, such as G-Box, Box 4, GATA-motif, GT1-motif, I-box, TCT-motif, AE-box, ATCT-motif, chs-CMA1a/2a/2b/2c, TCCC-motif, ACE, and LAMP-elements. Notably, the most prevalent element in the light-responsive category was Box-4, constituting 23%, followed by G-Box at 17%. In contrast, AC-I and AAAC-motif were the least represented, each accounting for only 1% of the elements in this category (Fig. 5d). Within the stress-responsive category, various *cis*-elements were identified, including MBS, ARE, WUN-motif, TC-rich repeats, LTR, CCAAT-box, MBSI, Myb-binding site, GC-motif, and TATC-box. Notably, ARE constituted the highest percentage at 42%, followed by WUN-motif, LTR, and MBS, each representing 12%. In contrast, TATC-box had a minimal presence, accounting for less than 1% of the elements in this category (Fig. 5e). In terms of regulatory elements associated with the response to stress and hormones, it should be noted that many of these factors overlap, as many hormones are induced in response to stress. Therefore, the *CER* transcript profiling may differ for hormones and stress responses.

### Protein-protein interaction and *GhCER* genes-transcription factor regulatory network

**Fig. 6:**
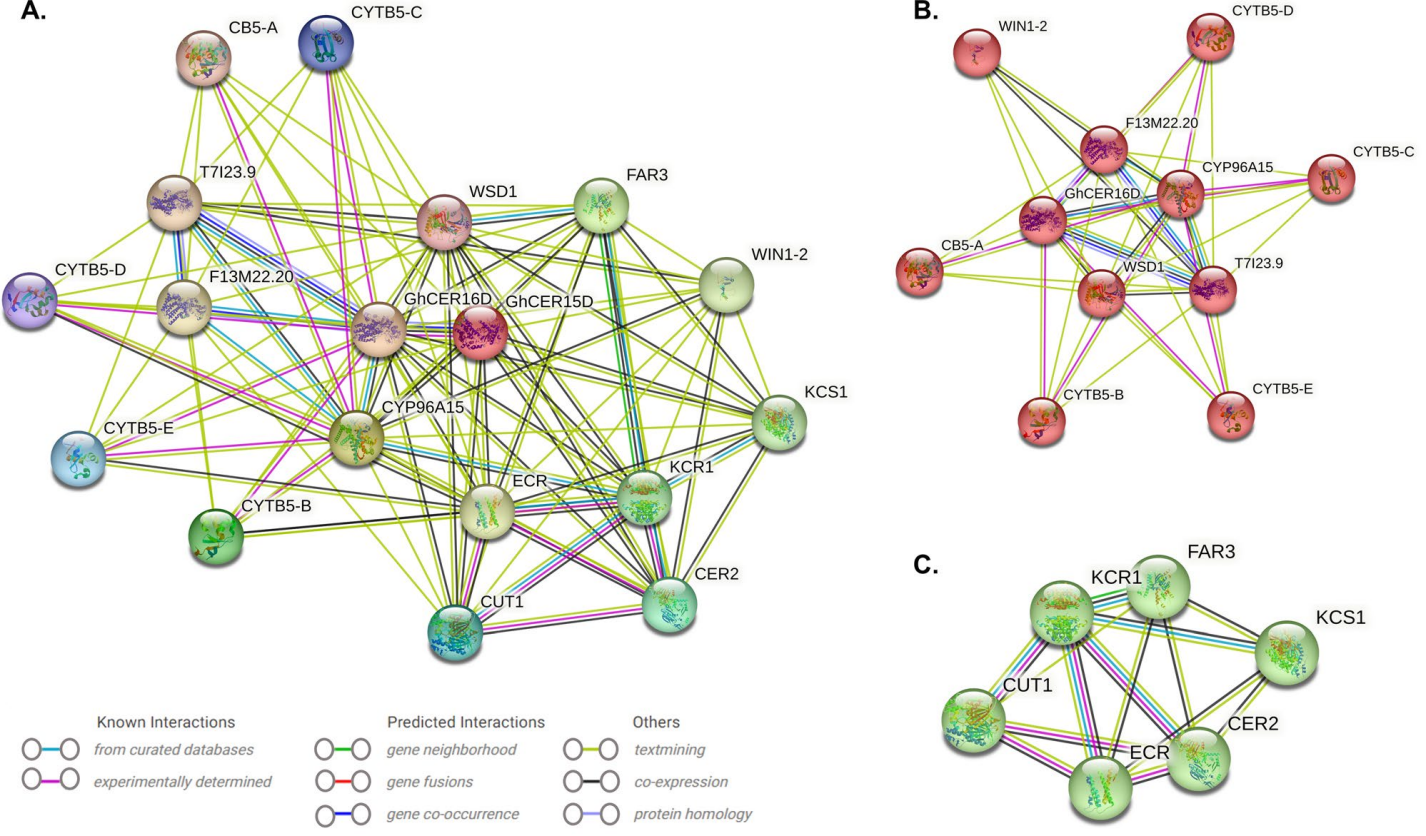
**A.** Shows the predicted regulatory protein-protein interaction network of CER. The network was created using the online software STRING. The proteins were represented at the network nodes with 3D structures of the proteins in the nodes, and the line colors indicate different data sources. **B** and **C** represent different clusters

The protein-protein interaction network for CER proteins was established using orthologous proteins from *Arabidopsis*. GhCER proteins exhibiting the most pronounced homologous similarity to their *Arabidopsis* counterparts were chosen as STRING proteins. All 16 GhCER proteins are connected to recognized *Arabidopsis* proteins in the network (Fig. 6a, Table S5). GhCER proteins categorized into distinct groups likely serve varying functions. GhCER01A, GhCER02A, GhCER03A, GhCER04A, GhCER05A, GhCER11D, and GhCER06A were homologous with AtCER1; GhCER07A, GhCER08A, GhCER09A, GhCER13D, GhCER14D, GhCER15D, and GhCER10D with AtCER3; and GhCER16D with AtCER10 and *AtCER1*, and they all had strong interaction with each other (Fig. 6a). Next, we clustered our network to find out whether there are more interactions among them or whether the proteins are at least partially biologically connected. Three main clusters were identified: Cluster 1 contains CB5-A, CYP96A15, CYTB5-B, CYTB5-C, CYTB5-D, CYTB5-E, F13M22.20, GhCER16D, T7I23.9, WIN1-2, WSD1 and is mainly associated with the process of wax synthesis. The second and third clusters contain CER2, CUT1, ECR, FAR3, KCR1, KCS1 and GhCER15D, which are mainly involved in lipid metabolism (Fig. 6b and c). GhCER proteins exhibit robust interactions with recognized Arabidopsis proteins, suggesting potential functional similarities to their counterparts in *Arabidopsis*. The thickness of the line between proteins corresponds to the strength of their interaction, with a higher interaction coefficient resulting in a thicker line, and vice versa (Fig. 6, Table S5).

**Fig. 7:**
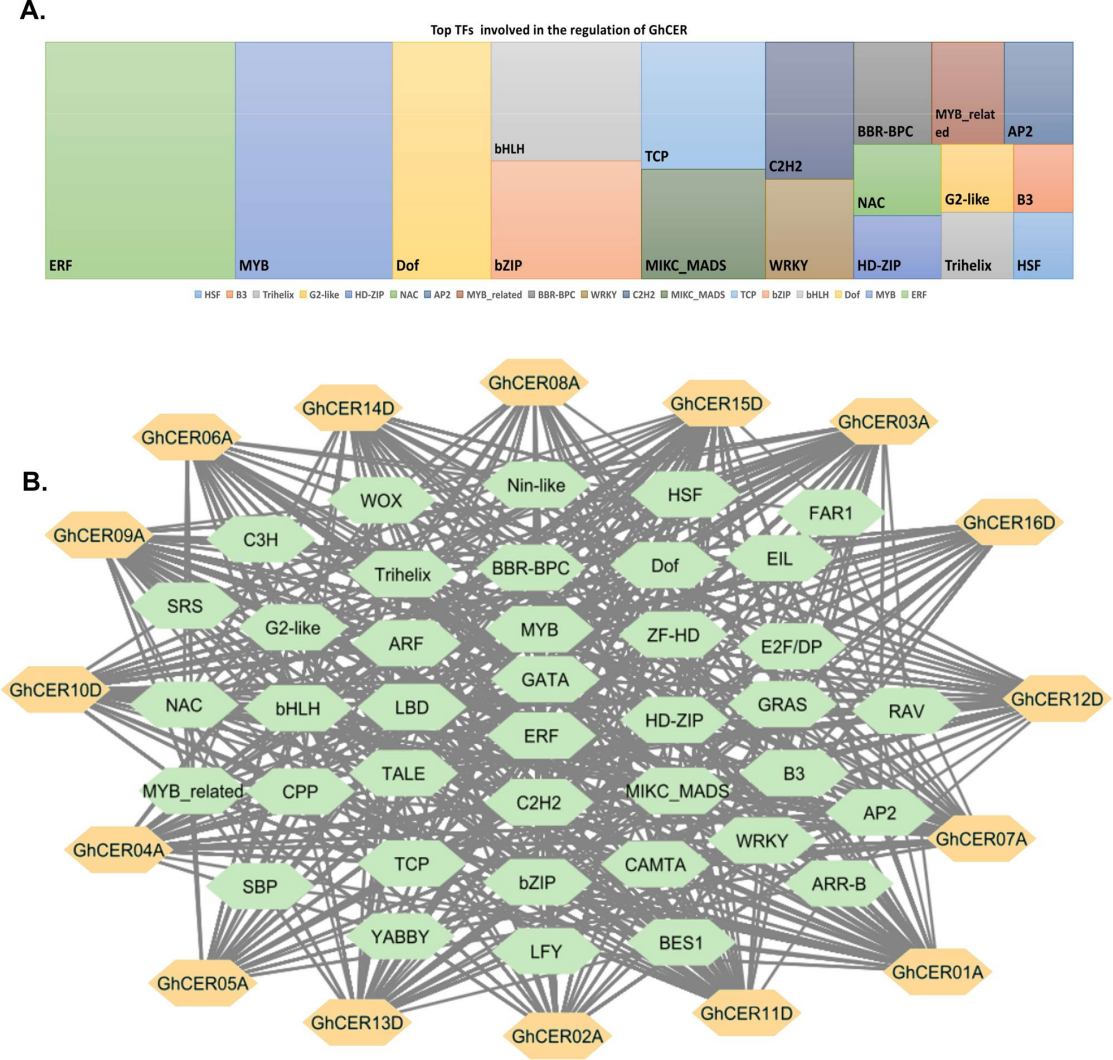
Putative transcription factor regulatory network analysis of CER genes. **(A)** Top TFs involved in the regulation of *GhCER* genes. **(B)** Green hexagonal nodes represent transcription factors; orange hexagonal nodes represent *GhCER* genes, and node size represents the degree of interaction between nodes based on the degree value

Furthermore, the prospective regulatory transcription factor network for *GhCER* genes was determined by retrieving the 2,000 bp sequences upstream of all 16 *GhCER* genes and subjected to analysis utilizing the PTRM online database. The results revealed that 3402 TFs from numerous TF families, namely ARF, AP2, bHLH, Dof, bZIP, E2F/DP, ERF, GATA, MYB, NAC, SBP, TCP, WOX, and WRKY, were associated with the control of the 16 *GhCER* genes (Table S6). Within the identified transcription factors (TFs), the ERF family exhibited the highest frequency with 574 members, followed by the MYB family with 476 members. Other prominent TF families included Dof (297), bHLH (229), bZIP (226), and TCP (202). Conversely, the least represented TF families were ARR-B (3), E2F/DP (3), and C3H (Fig. 7a, Table S6). All *GhCER* genes were targeted by multiple TF family members, with *GhCER*15D and *GhCER*11D interacting with 32 TF families, *GhCER*01A with 31 and *GhCER*13D, *GhCER*07A and *GhCER*10D with 27 TF families. Notably, the *GhCER*15D gene stood out as the one targeted by the highest number of TFs (375), followed by *GhCER*09A (340) and *GhCER*03A (255). The regulatory TF network of the five most frequently targeted *GHCER* genes is shown in Fig. 7b. This investigation also anticipated the involvement of various transcription factor (TF) families, such as bHLH, ERF, MYB and AP2, in fatty acids and wax biosynthesis. In addition, numerous transcription factors associated with the growth and development of plants, comprising bHLH, TCP, WRKY, BBR-BPC, AP2, and LBD were recognized in the *GhCER* genes. Significantly, ERF and AP2 TFs were widely distributed across a substantial proportion of *GhCER* genes (Table S6).

### Prediction of prospective *CER* gene-targeting mirnas across the genome

**Fig. 8:**
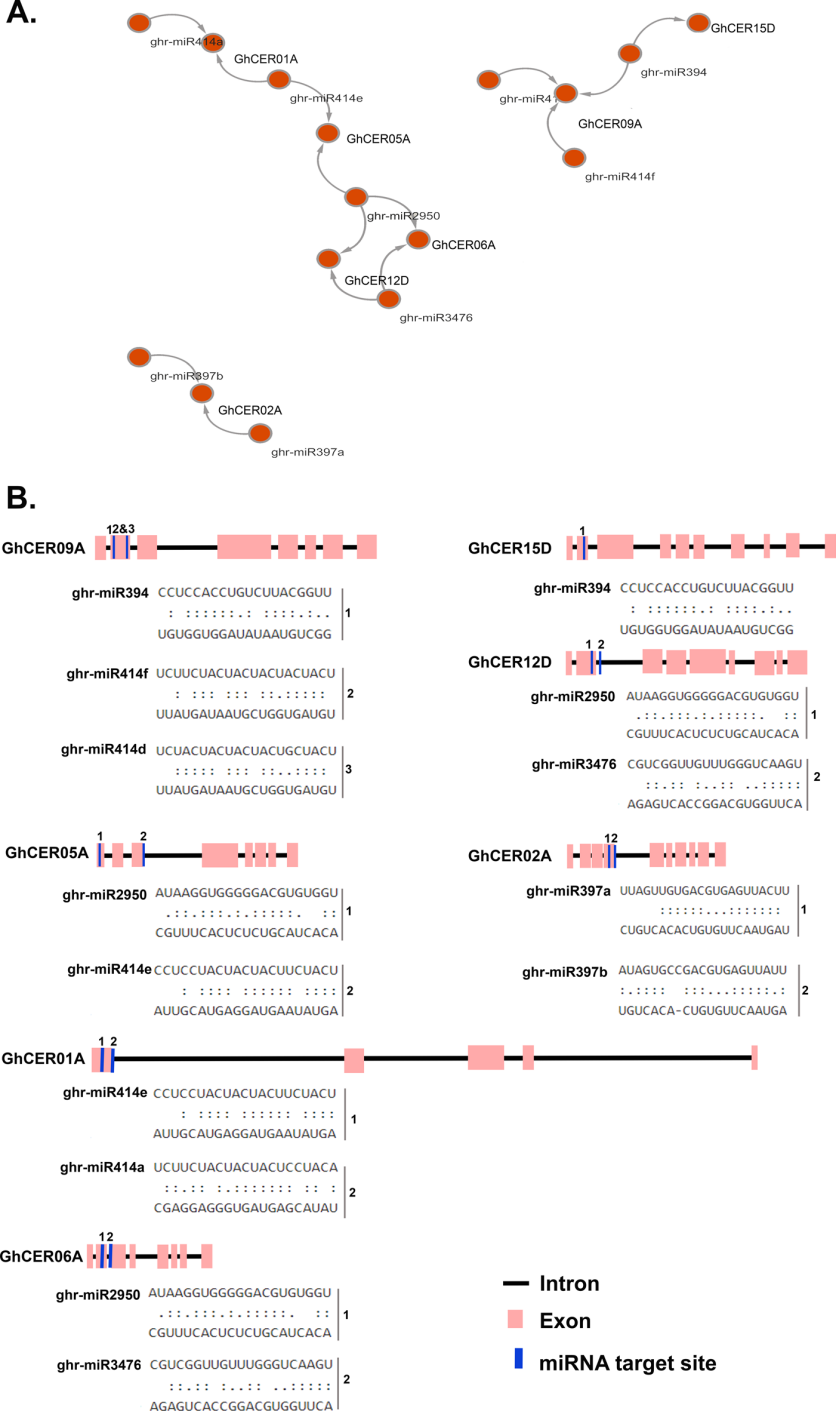
Predicted miRNA targeting *CER* genes. **(A)** Network representation of predicted miRNA-targeted CER genes. **(B)** Schematic representation of *CER* genes targeted by miRNAs

To enhance our comprehension of the regulatory processes involving miRNAs in the modulation of *GhCER* genes, we identified nine potential miRNAs targeting seven *GhCER* genes, as illustrated in the network diagram (Fig. 8a) and the schematic representations of *GhCER* genes targeted by miRNAs (Fig. 8b). Comprehensive details regarding the supposed miRNA target sites and corresponding *GhCER* genes can be accessed in Table S7. The findings revealed that four members of the *ghr-miR414* family target three genes: *GhCER*05A, *GhCER*01A, and *GhCER*09A. *GhCER*02A is targeted by two members of the ghr-miR397 family; all three genes *GhCER*12D, *GhCER*06A and *GhCER*02A are targeted by the *ghr-miR2950* family; the *ghr-miR3476* and *ghr-miR394* families were also involved in regulating the expression of *GhCER*15D and *GhCER*09A, respectively. In addition, the *ghr-miR394* and ghr-miR414 families both targeted the *GhCER*09A gene. Two miRNA families, *ghr-miR2950* and *ghr-miR3476* targeted the *GhCER12D* gene but *GhCER15D* was only targeted by one, *ghr-miR394* (Fig. 8a and b, Table S7).

Overall, three distinct interaction patterns between mRNA and miRNA were uncovered, encompassing 3’-compensatory sites, 5’-dominant canonical sites, and complementary sites. Approximately 28% of the miRNAs are intronic (in-miRNAs), while 65% are exonic, and their production is directly linked to the transcription of the respective host genes. Additional investigation is needed to explore the expression levels of these predicted miRNAs and their associated target genes, as well as to elucidate their functional roles in cotton (Fig. 8b).

### Enrichment analyses of *GhCER* genes using GO and KEGG pathway analysis

**Fig. 9:**
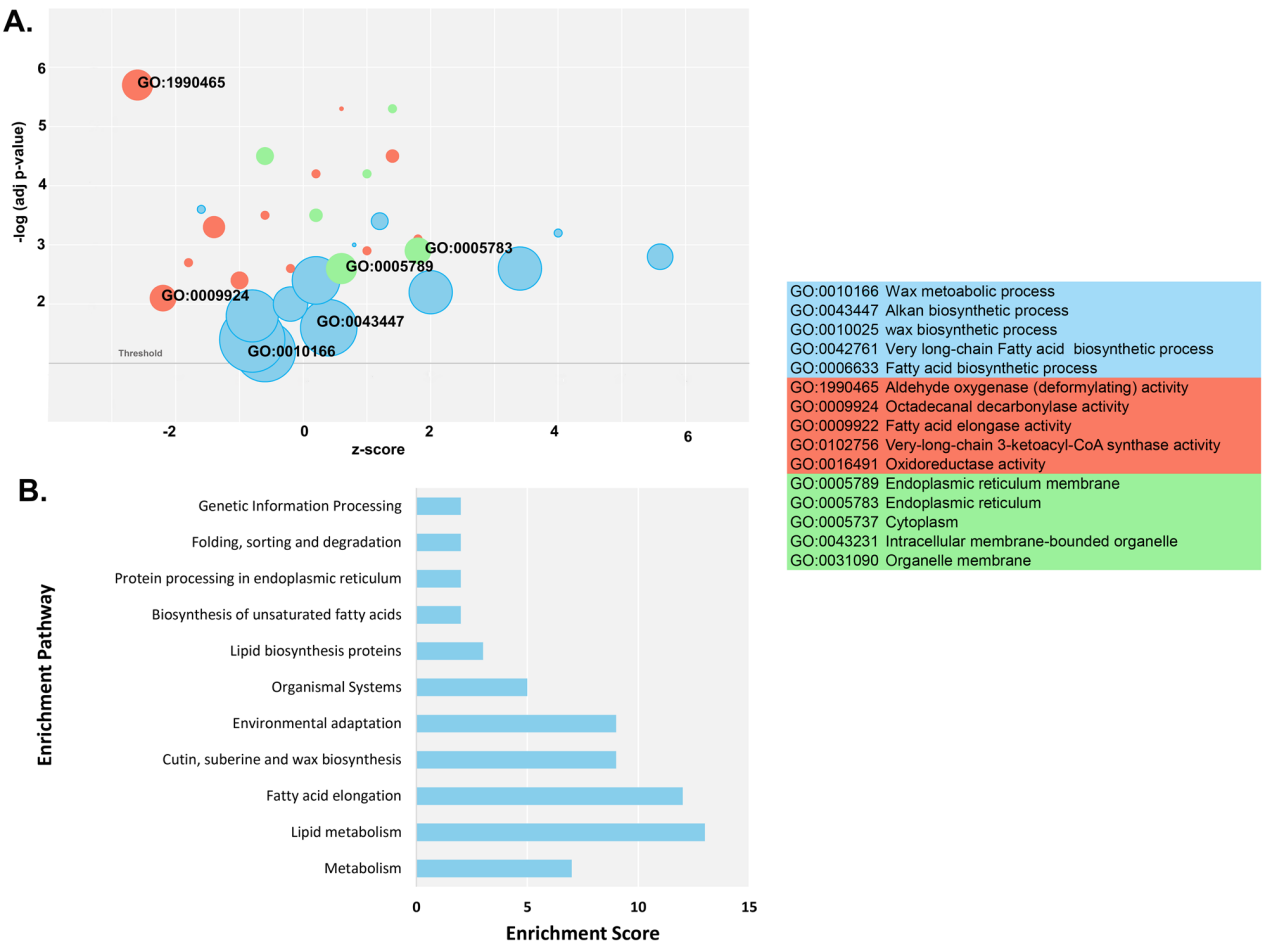
Gene Ontology (GO) and Kyoto Encyclopedia of Genes and Genomes (KEGG) enrichment analyses of CER genes **(A)** Highly enriched GO terms in *CER* genes. **(B)** Highly enriched KEGG pathways in *CER* genes

Enrichment analyses utilizing Gene Ontology (GO) and Kyoto Encyclopedia of Genes and Genomes (KEGG) were conducted for the *GhCER* genes, and the outcomes were categorized into three distinct classes: cellular component (CC), molecular function (MF) and biological process (BP) Table S8a provides a comprehensive annotation of the enriched terms within each class. It is noteworthy that the GO-BP class has the highest enrichment with 13 terms. This was followed by the GO-MF class with 12 enriched terms and the GO-CC class with six enriched terms (Fig. 9a, Table S8a). In the GO-BP class, the three most enriched terms include alkane biosynthesis (GO:0043447), wax metabolism and biosynthesis processes (GO:0010166, GO:0010025), fatty acid biosynthesis processes (GO:0006633) and response to abiotic stimuli (GO:0009628). GO-MF enrichment includes five highly enriched terms, such as aldehyde oxygenase (deformylating) activity (GO:1,990,465), octadecanal decarbonylase activity (GO:0009924) and fatty acid elongase activity (GO:0009922). Within GO-CC, the most enriched terms are endoplasmic reticulum (GO:0005789), cytoplasm (GO:0005737) and intracellular membrane-bound organelle (GO:0043231).

KEGG pathway analysis revealed 17 predicted pathways for the 16 *GhCER* genes. Figure 9b shows the highly enriched pathways, including fatty acid elongation (00062), lipid metabolism (B09103), suberin, cutin and wax biosynthesis (00073) and environmental adaptation (B09159) (Fig. 9b, Table S8b). Overall, GO and KEGG enrichment analyses show that *GhCER* genes may be involved in a variety of molecular, cellular, and biological processes like plant metabolism, wax and fatty acid synthesis, and responses to both biotic and abiotic stressors.

### Expression profiles of *GhCER* genes across various tissues in upland cotton

**Fig. 10:**
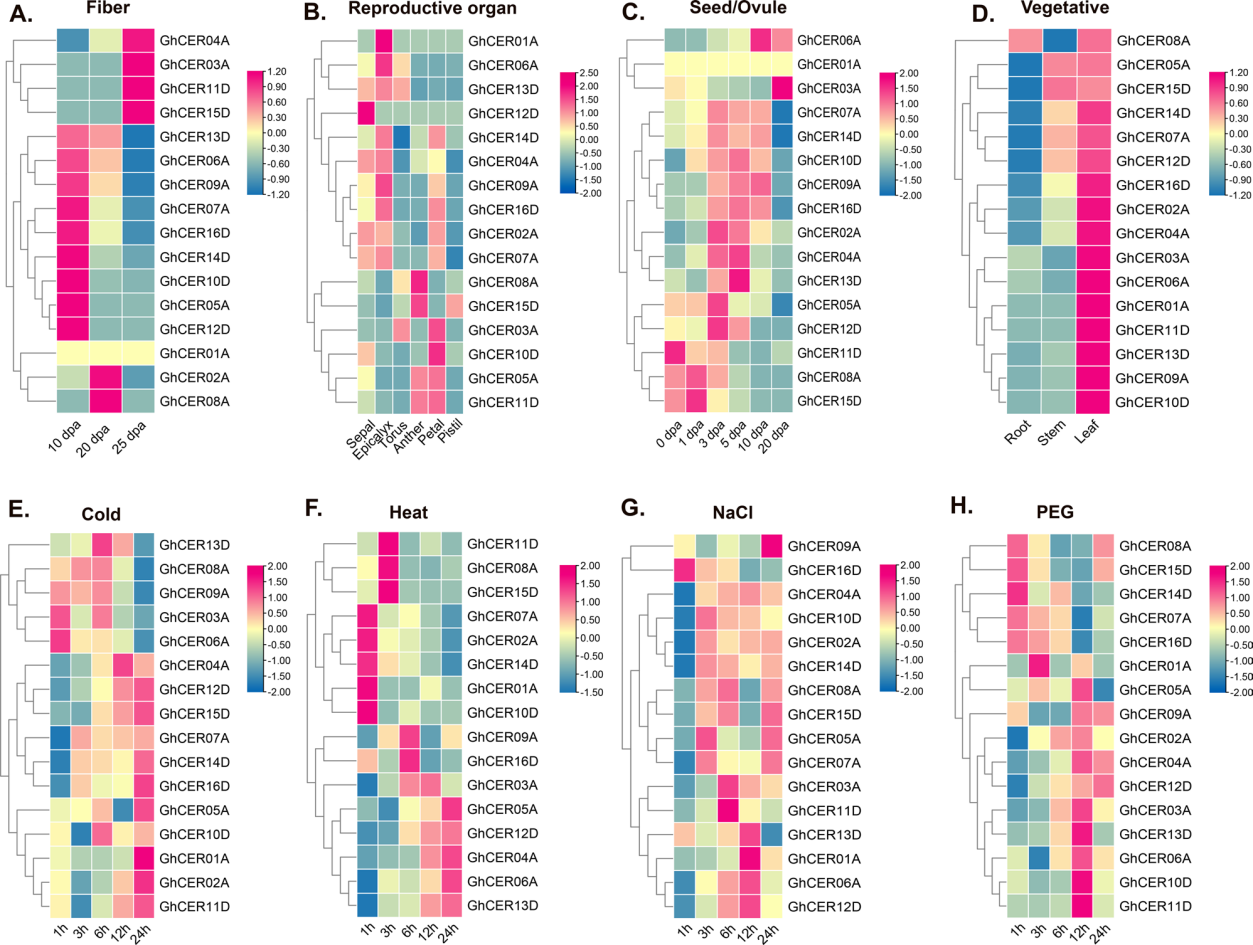
Expression profile of 16 *GhCER* genes in upland cotton. **(A)** A heat map showing the expression profile of GhCER genes in different tissues of cotton. **(B)** Expression pattern analysis of GhCER genes in response to abiotic stress. FPKM values were log2 transformed and the heatmap was constructed using TBTools software. The red colour shows the highest and the blue colour shows the lowest expression levels in the expression bar

To determine the likely involvement of the 16 *GhCER* genes in growth and development, researchers examined their expression patterns in various tissues, including cotyledon, calycle, leaves, petals, pistils, root, stamen, stem, tours, ovule, fibre, and seed, using publicly transcriptome data from the Cotton Omics Database (http://cotton.zju.edu.cn/) (Fig. 10a-d). Five *GhCER* genes (*GhCER02A*, *GhCER07A*, *GhCER10D*, *GhCER13D* and *GhCER14D*) were overexpressed in almost all tissues and at all developmental stages. *GhCER13D* and *GhCER14D* were similarly expressed in leaves, roots and stems during ovule development. *GhCER01A* was only expressed in leaves and epicalyx. In contrast to the vegetative developmental stage, *GhCER02A* showed relatively higher expression in other tissues, especially in fibres and ovules. These findings imply that the majority of *GhCER* genes have a greater probability of being expressed in vegetative tissues.

### Expression profiling of *GhCER* genes in response to abiotic stress

We analyzed publicly available transcriptome data to determine the impacts of heat and cold stress, salt, and PEG treatment to investigate the putative relevance of *CER* genes in the response of upland cotton to abiotic stress (Fig. 10e-h). Significant changes in the expression patterns of most *GhCER* genes were observed after abiotic stress treatment. *GhCER04A* and *GhCER12D* showed up-regulation after cold stress, PEG and NaCl treatment, especially 12 h and 24 h after treatment. *GhCER14D*, on the other hand, was stimulated by elevated temperature, cold, and salinity, with expression peaks detected at 1 h, 6 h, 12 h, and 24 h. Nevertheless, their expression exhibited a decrease following exposure to 4 °C and PEG. These results suggest that *GhCERs* are involved in the response to abiotic stress.

**Fig. 11:**
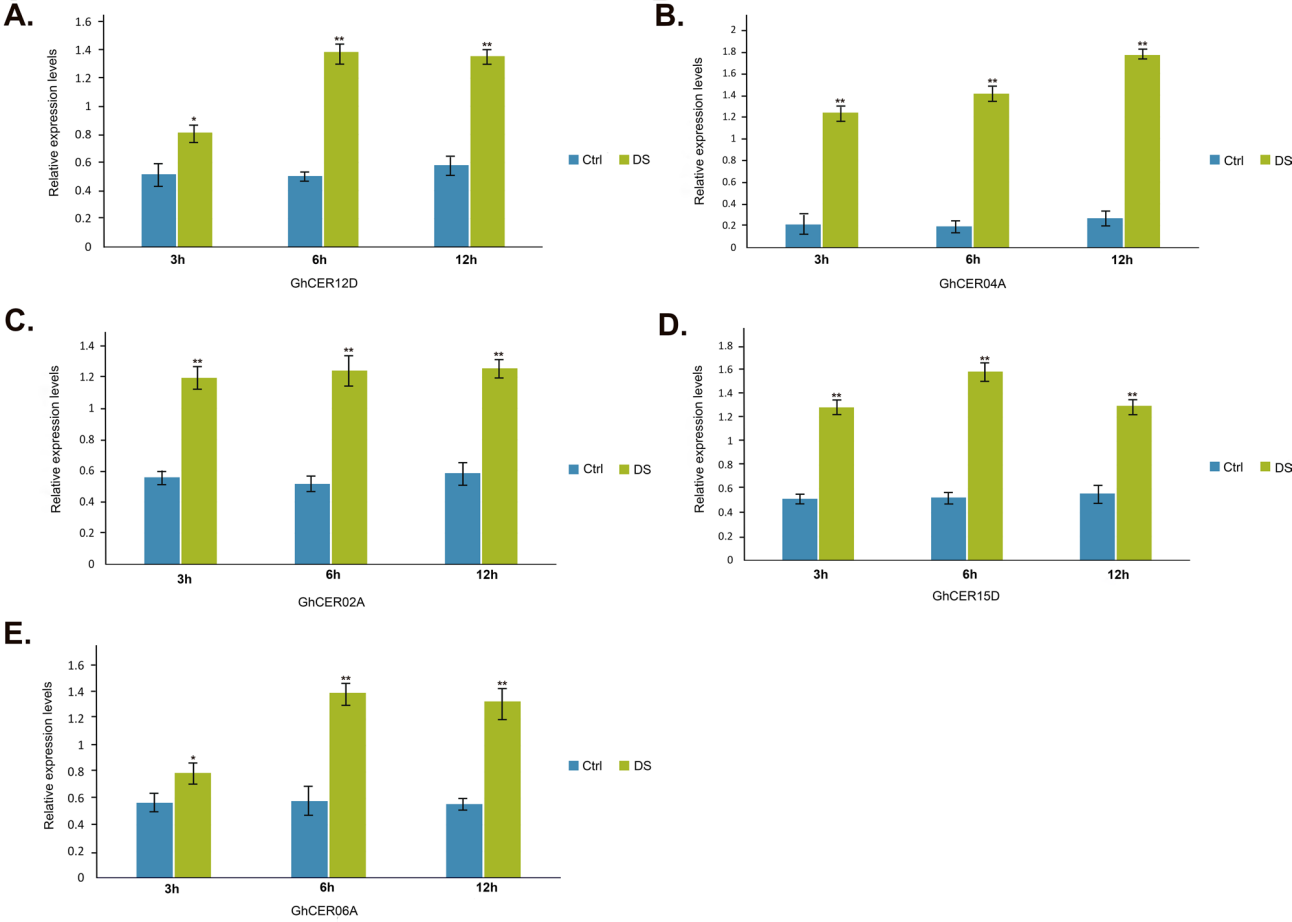
Relative expressions of *GhCER* genes. qRT-PCR analysis was performed to observe the relative expression patterns of *GhCER* genes at 3 h, 6 h and 12 h under control and drought stress conditions. Vertical bars represent mean ± SD (*n* = 3). * and ** indicate significance at *p* ≤ 0.05 and *p* ≤ 0.01, respectively

Subsequently, utilizing significantly different FPKM expressions, *cis*-elements, and enrichment analyses, we examined the gene expression patterns of the five *GhCER* genes when subjected to drought stress at the seedling stage. This study was performed on leaves of G. *hirsutum* seedlings exposed to drought stress (SD) for 12 h and 24 h, and the results were analysed using a qRT-PCR assay (Fig. 11). We found that the transcription of *GhCER12D*, *GhCER06A* and *GhCER02A* was higher in the drought-stressed genotype than in the controls. The relative expression analysis revealed that the *GhCER15D* expression was six times more in the leaves under drought stress than in the control conditions. The high expression of *GhCER04A* and *GhCER12D* under drought stress indicates that tolerant genotypes have higher cuticular wax production under drought stress, which is due to their role in wax biosynthesis. These results suggest that these genes are linked with the synthesis of cuticular wax under conditions of water scarcity.

### Cotton’s drought resilience is reduced when *GhCER04A* is silenced

**Fig. 12:**
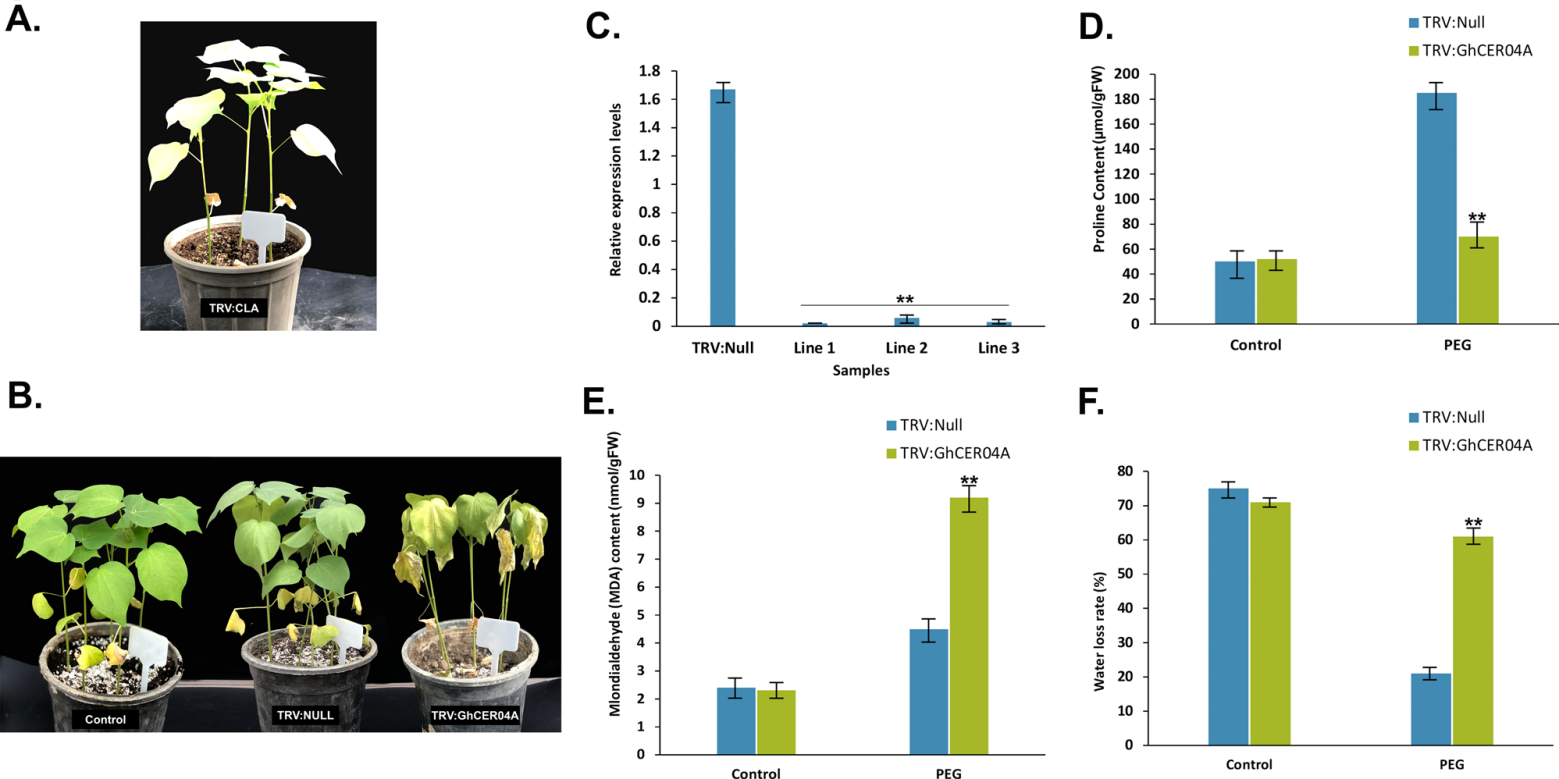
Silencing of the *GhCER04A*. **A** gene reduced the tolerance of cotton plants to drought stress. A Plant with albino phenotype (TRV: GhCLA1, positive control). **B** Phenotype of the negative control (TRV: null) and the transgenic plants with silenced *GhCER04A* gene (TRV:*GhCER04A*) under drought conditions for 48 h. **C** Relative expression of *GhCER04A* in the control plants (TRV: null) and the silenced plants (TRV:*GhCER04A*). The *GhUBQ7* gene was used as an internal control. **D-F** Physiological indicators were measured on plants grown under control and drought conditions. **D** proline content; **E** malondialdehyde (MDA) content; (**F**) water loss rate; error bars indicate the standard deviation from three independent experiments

RNA-Seq study revealed that the *GhCER04A* gene expression showed a persistent rise in response to PEG treatment during a 12-hour time window. We used functional analysis through the virus-induced gene silencing (VIGS) approach to characterize the putative role of the *GhCER04A* gene, under drought stress conditions in upland cotton. After a 10-day inoculation period, the TRV: CLA (positive control) plants exhibited an albino phenotype, indicating successful gene silencing (Fig. 12a). Two weeks after infection, both TRV: NULL and TRV:*GhCER04A* plants were exposed to drought stress. The TRV: *GhCER04A* plants showed a distinct phenotype with wrinkled leaves under drought conditions compared to the control plants (Fig. 12b). The quantitative real-time PCR (qRT-PCR) results verified that the expression level of the plants with silencing (TRV:*GhCER04A*) was significantly reduced compared to the negative control (TRV:00) (Fig. 12c).

To investigate the effects of *GhCER04A* on drought stress, changes in physiological metrics, including malondialdehyde (MDA), water loss rate, and proline, were examined after 12 h of exposure to 20% PEG6000. After drought stress, proline levels (Fig. 12d) were significantly higher in *GhCER04A*-expressed lines than in *GhCER04A*-suppressed lines, while MDA content and water loss rate were significantly lower than in *GhCER04A*-suppressed lines (Fig. 12e&f). These findings imply that suppressing the *GhCER04A* gene may reduce cotton plants’ physiological tolerance to drought stress.

## Discussion

The plant cuticle is a layer of hydrophobic material that serves as the initial barrier separating the plant from its surroundings, thus shielding it from abiotic and biotic stress. It consists of VLCFAs and derivatives [36]. Cuticular wax production is regulated by several genes, including *CER* [9], *KCR* (beta-ketoacyl-CoA reductase) [37], *KCS* [38], *FAR* (fatty acid CoA reductase) [39], *CUT1* [40], FAE (fatty acid elongase) [41], and *LACS* [42]. The *CER* gene family contributes to the production of cuticle wax and has a vital function in stress mitigation [12]. The *CER* gene family has been found across the genome in a range of plant species, including *Castanea mollissima* [32], apple [29] and passion fruit (*Passiflora edulis*) [31], and functionally described in (A) *thaliana* [43], Barley [44], *Brachypodium distachyon* [45], and (B) *napus* [46]. However, the *CER* genes in cotton (*G. hirsutum*) have not yet been studied. In this investigation, we carried out a detailed study of the *CER* genes from four cotton species.

Sequence alignment enabled us to identify 52 genes of the *CER* family in four species. The chromosomal arrangement analysis revealed that the cotton *CER* genes are randomly distributed across the chromosomes. Within G. *hirsutum*, the distribution of 16 *CER* genes was uneven across ten chromosomes, with the predominant allocation occurring on chromosome A06, as delineated in Table S1. In G. *barbadense*, the *CER*s were located on ten chromosomes and one scaffold. In both G. *raimondii* and G. *arboreum*, the identified *CER*s were spread across the five chromosomes, but the genes were located on different chromosomes in each species. The occurrence of genes from the same family on different chromosomes indicates the diverse range of functions carried out by these genes [47]. The *CER* gene count in cotton species was similar to that in apples (ten genes) [48], whereas some species such as sunflower, have more *CER* genes, (thirty-seven) [30], and jujuba *CER* (twenty-nine) [28], the variation in the gene number could be credited to variation in genome size. Using the protein sequences of G. *arboreum*, G. *hirsutum*, G. *barbadense*, G. *raimondii*, and A. *thaliana*, a phylogenetic tree was built and stratified into six clades (Fig. 1). The diploid and tetraploid CER proteins of *Gossypium* species and A. *thaliana* were clustered together, but for some of the CER proteins of A. *thaliana*, they were classified separately in a single group, suggesting that the *CER* genes of cotton may have a different function from the *CER* genes of A. *thaliana*. Many reports suggest that genes located in the same phylogenetic group may have similar functions [49, 50].

The identification of common motifs during gene family evolution can be used to explain differences in protein function [51]. Also, we expected conserved motifs within the *GCER* proteins and discovered a diverse motif arrangement ranging from 1 to 15 motifs. This suggests a notably conserved protein structure among GCER proteins. Our findings differ from those of Ahmad et al. (2021), who observed similar motifs in sunflower. Discrepancies in the exons and introns number represent a significant source of diversity in gene families, influencing gene function and expression [52]. In this study, *CER* genes were found to have quite different numbers of introns, ranging from 3 to 13 introns, which is less than passion fruit (1–28) [31], and sunflower (1–19) [30]. The *GaCER09* gene has a lower number of introns (2), while *GaCER08* contains 11 introns. Moreover, the intron/exon structure of each gene was positioned in alignment with its corresponding location on the phylogenetic tree. These results suggest that the ancestors of *GCER* genes may have undergone numerous rounds of intron loss and gain during evolution, facilitating alternative splicing and leading to the production of messenger RNA and protein isoforms with different functions [53, 54].

Examination of the *cis*-regulatory elements of *CER* genes showed that ERE, ABRE, GARE motif, TCA, ARE, G-box, Box-4 and GATA *cis*-elements were abundant, suggesting that *CERs* may be associated with a variety of developmental and stress responses in plants [11, 15, 31]. AP2/ERF elements have a significant role in abiotic stress responses in plants [55, 56]. GARE motifs respond to gibberellin and have a vital role in orchestrating stress regulatory networks, especially under salt and water deficit stress [57]. ABRE elements participate in conferring drought stress tolerance and necessitate abscisic acid (ABA) for full activation [58] TCA elements, on the other hand, are implicated in the salicylic acid stress response process [59]. The G-box serves as a crucial element the plant’s reaction to abiotic stress factors [60]. GARE *cis*-elements are involved in the hormone response [61]. Yu et al. (2021) described the participation of GATA and G-box elements in abiotic stress responses and hormone signalling involved in defense [62].

Replication events emerge as a key factor driving the evolutionary diversification of genomes and genetic systems [63]. The count of gene family members was impacted by environmental factors and artificial selection. The gene sequences in cotton underwent duplication through repeated occurrences, leading to the selective loss or recombination of some redundant genes [64]. The total number of *SBT* genes of G. *hirsutum*, was less than the sum of the genes of G. *raimondii* and G. *arboreum*, [65], and after polyploidization, the gene number in G. *hirsutum SAC* gene family members decreased [66]. In our research findings, the *CER* gene number in G. *hirsutum* and *G. barbadense* was lower than the combined total in G. *raimondii* and G. *arboreum*. Allotetraploid genomes experienced lower selection pressure compared to diploid genomes [67]. Previous research indicate that purifying selection was the primary evolutionary factor at work on the *CER* gene [30]. Consistent with this notion, our results indicated that more than 90% of *CER* gene pairs across the four cotton species consistently exhibited Ka/Ks ratios less than one. This shows that *CER* was subjected to purifying selection during cotton’s evolution.

The network of protein-protein interactions within a gene family serves as evidence for the connections between the individual members of the family [68]. The results of GhCER protein-to-protein interactions showed that six GhCER proteins are homologous to AtCER1. AtCER1 actively participates in wax biosynthesis and demonstrates dynamic responsiveness to both biotic and abiotic stressors [69]. Six GhCER proteins exhibit homology with AtCER3, and previous research has demonstrated that *AtCER3* interacts with AtCER1, facilitating the catalysis of redox-dependent Very Long-Chain Alcohols (VLCAs) from very long-chain acyl-CoAs [43]. Some GhCER proteins exhibit AtECR homology and interact with AtCUT1, AtCYP96A15 and *AtWIN1*-2. According to Zhukov et al. (2018), AtECR (very-long-chain enoyl-CoA reductase) is involved in the production of VLCFAs of varying chain lengths, which are involved as membrane precursors in numerous biological processes [70]. AtCUT1 encodes an enzyme that condenses very long-chain fatty acids and is involved in the biosynthesis of cuticular wax and pollen fertility [71]. The ethylene-responsive transcription factor WIN1 enhances cuticle development by increasing the expression of enzymes involved in wax production and confers drought tolerance [9]. Some GhCER proteins show sequence homology with AtKCS. According to Tong et al. (2021), AtKCS plays a role in the biosynthesis of very-long-chain fatty acids (VLCFAs) and is vital for the synthesis of cuticular wax and suberin [72]. The results of the analysis of protein-protein interactions between GhCER proteins imply that the homology and interaction with well-characterized *Arabidopsis* proteins may indicate common functionalities, including involvement in plant development, wax biosynthesis and stress response. However, additional investigation is required to attain a more comprehensive understanding.

Recently, several miRNAs involved in various metabolic processes, development and environmental stresses have been identified in multiple plants [33, 73, 74], in Arabidopsis, miRNAs such as trans-acting small interfering RNA (tasiRNA) were shown to be involved in *CER3* silencing during stem wax synthesis [75]. likewise, Liu et al. (2019) suggested that Brassica miRNA (*bna-miR165a-5p*) may be involved in wax formation by modulating the expression of the gene *BnaA06g40560D* [76]. These results showed that miRNA could play an essential role in wax production by specifically regulating genes. In this study, we identified 9 putative miRNAs targeting five *GhCER* genes, with three members of the *ghr-miR414* family targeting three *GhCER* genes. It’s noteworthy that *miR414* has been previously found to be associated with drought stress in barley [77] and rice [78], pollen development of *Brassica rapa* [79], disease resistance in aubergine (*Solanum melongena* L.) [80], flower development in *Hypericum perforatum* L. [81]. , fibre formation [82], and in salt stress in cotton [83]. A member of the *ghr-miR2950* family targets three *GhCER* genes, and *miR2950* has been observed to be involved in the response to Cotton leaf curl multane virus [84]. The *ghr-miR3476* and *ghr-miR394* families each target two *GhCER* genes. *miR394* is associated with the response to salt and drought stress [85], cold stress [86] in *Arabidopsis* in an abscisic acid-dependent manner and suppresses leaf curl in rice [87]. These findings imply that the discovered *ghr-miRNAs* may play an important role in the response to diverse stress conditions in cotton by influencing the transcription levels of *CER* genes. However, more research is required to validate this concept.

Plant TFs have been shown to regulate wax production and fatty acid as well as stress responses in various situations [88]. In this study, we predicted various transcription factors (TFs) targeting the 16 *GhCER* genes and established their regulatory network connections. The ERF transcription factor family was the most prevalent, followed by MYB, Dof, bHLH, bZIP and TCP. This indicates their possible involvement in the promotion or inhibition of wax and biosynthesis of fatty acid as well as in the response to various stress factors. Drought resistance in apple is conferred by the AP2/EREBP transcription factor, which positively regulates wax production [89]. The AP2/EREBP transcription factor zmereb160 of maize increases the drought tolerance of *Arabidopsis* by increasing the amount of wax on the leaves [90]. bHLH transcription factors are associated with the biosynthesis of cuticular wax and tolerance to drought stress in *Helianthus annuus* L. [91]. TCP TFs control growth and development of plants and stress response [92]. MYB TFs modulate the biosynthesis of VLCFA, metabolism, plant development, and stress response [93]. Li et al. [42] reported that *MaMYB13* is associated with the cold stress response by activating the gene expression for the VLCFAs and phenylpropanoids biosynthesis in banana fruit after harvest [94]. bZIP transcription factors have an essential role in the life cycle of plants, encompassing functions such as pathogen defense, stress response, seed maturation, secondary metabolism and development of flower [95]. Our results are consistent with previous studies and suggest a possible link between the function of transcription factors (TFs) in *GhCER* genes and the regulation of stress resistance in plants. However, further investigation is needed to comprehensively understand their role in wax regulation.

The *GhCER* genes were also subjected to GO and KEGG annotation analysis. Most GO terms were associated with wax and lipid metabolic processes, endoplasmic reticulum, fatty acid synthase, Cuticle development and response to abiotic stimulus. The KEGG pathway that was enriched most among *GhCER* genes include those involved in metabolism of lipid and fatty acid, suberin, wax and cutin production, along with pathways related to environmental adaptation. Our findings align with previous research on passion fruit (*Passiflora edulis*), which also identified similar Gene Ontology (GO) terms and KEGG pathways associated with plant-pathogen interaction, biosynthesis of wax and fatty acid, and various stress responses [31].

The expression profiling results indicate that *GhCER* genes exhibit differential and constitutive expression patterns across various cotton tissues under *distinct* conditions (Fig. 10a and b). The FPKM expression results revealed higher expression levels of *GhCER* genes in the leaves of plants subjected to drought-stress when compared to well-watered plants, which is consistent with the qRT-PCR results indicating increased *GhCER* gene expression in the leaves of the drought-stressed plant when compared to the non-stressed plant group. Likewise, Gao et al. [29] found that there was higher *MdCER1*-1 expression in drought-stressed leaves when compared to leaves from irrigated plants [29]. The study by Wu et al. [21] also highlighted that *SlCER1*-1 induces wax alkanes synthesis, which improves drought resistance and fruit storage. In addition, the FPKM expression results showed differential expression between different tissues and *GhCER* genes, such as *GhCER01A*, which was only expressed in leaves but showed no expression level in other plant tissues. Similar findings were reported by Pascal et al. [96], indicating that the *CER26* expression was not prominent in stems but high in leaves, *CER2* exhibited expression in all tissues, and *CER26*-like genes showed strong expression in flowers but only marginal expression in all other tissues [96]. The study by Rizwan et al. [31] similarly observed different expression patterns of *PeCER* in various tissues, with *PeCER1*, *PeCER11*, *PeCER15*, and *PeCER32* showing strong expression in roots but very low expression stem tissues [31]. Furthermore, the results of *GhCER* gene qRT-PCR expression were aligned with the expected results of transcription factors (TFs), indicating that the expression levels of *GhCER* genes harbouring multiple transcription factors (*EFRs*) for very long chain fatty acids (VLCFAs) and wax were elevated under stress conditions in contrast to control conditions. These observations indicate an important function of the *GhCER* genes, which either positively or negatively regulate responses under stress conditions. After conducting transcriptome analysis and qRT-PCR, we noticed a significant upregulation in the expression of the *GhCER04A* gene under drought stress conditions.

To investigate the role of *GhCER* in drought tolerance of cotton, *GhCER04A* was silenced. The results showed that silencing of *GhCER04A* impaired the resistance of cotton to drought stress. In addition, we measured various physiological indices (including MDA and proline content and leaf water loss rate) under drought stress. The experimental results showed that the proline content was lower in the silenced plants, while the MDA content was higher in the silenced plants than in the control plants under drought stress. The relative water content in the leaves of the silenced plants was also lower under drought stress than in the control plants. The higher MDA content in the VIGS-silenced cotton suggests that the ability to remove ROS may be impaired [12]. Similarly, previous studies reported that *CER* are related to drought resistance in plants by modulating the accumulation of ROS [29]. As a kind of osmotic substance, proline content is an important index reflecting the degree of damage to plant membrane lipid peroxidation by stress [97, 98]. In this study, drought stress induced the accumulation of proline in cotton, thereby improving the osmotic adaptability of the plant. The lower accumulation of proline in the VIGS-silenced cotton indicates that the osmotic adaptability of these plants was weakened by silencing of the *CER* gene. Under drought stress, plants often reduce the degree of stomatal opening to limit water loss in the cells [99, 100]. In this study, our data showed that the stomata of VIGS-silenced cotton plants opened wider than controls under drought stress, suggesting that silencing of *GhCER04A* affects stomatal closure and thereby increases plant water loss.

## Conclusion

In this study, a comprehensive analysis was conducted to identify 52 genes belonging to the *CER* family across four cotton species. These identified *CER* genes underwent scrutiny for various aspects including physicochemical properties, chromosomal mapping, evolutionary relationships, motifs, gene structures, protein-protein interactions, regulatory TF networks, *cis*-regulatory elements, syntenic analysis, enrichment analysis, and putative miRNA predictions. The *CER* genes displayed signs of expansion and purification selection. Additionally, we identified several transcription factors, such as ERF, bHLH, TCP, and MYB, which collectively form a regulatory TF network associated with *GhCER* genes. Different FPKM expression patterns of *GhCER* genes were observed in various plant tissues, such as stems, roots, leaves, and fibre tissues, and under various environmental conditions. The qRT-PCR results demonstrated a strong upregulation of *GhCER2*, *GhCER12*, and *GhCER15* under water deficit stress compared to well-watered conditions. This study particularly highlights the significance of the *GhCER04A* gene in enhancing cotton drought tolerance by promoting increased tissue water retention. The identified genes and regulatory networks provide valuable insights for further understanding and improving cotton’s response to environmental stresses.

## Materials and methods

### Cotton *GCER* gene family identification

The annotation and reference genome files for *G. barbadense* (V1.1) and *G. hirsutum* (V2.1) (tetraploid cotton species) and their common diploid ancestors *G. raimondii* and *G. arboreum* (V2.1) were retrieved from the CottonFGD genome database (https://cottonfgd.net/*)* [101]. The *Arabidopsis* Information Resource (TAIR) (https://www.arabidopsis.org/) database was accessed to retrieve all known *Arabidopsis CER* family (*AtCER*) protein sequence. The Pfam database (https://www.ebi.ac.uk/interpro/entry/pfam/) was utilized to extract the *GCER* gene family (Pfam number: PF12076) [102], and the hidden Markov model (HMM) was downloaded. The *GCER* protein domain sequences were extracted from the protein data of four cotton species using BLASTP alignment and HMMER3.0 software. To screen the potential protein sequences, the E-value was set to 1e^− 20^. To increase the credibility of our results, we subjected the obtained candidate protein sequences to additional confirmation by uploading them to the Pfam database, SMART database (http://smart.embl-heidelberg.de/) [103], and conducted a CDD search using the NCBI database [56]. The ExPASy Proteomics Server software (http://www.expasy.org) was used to analyse the amino acid sequences of the *GCER* genes from all four cotton species [104]. Subsequently, calculations were performed to determine the isoelectric point (PI) and amino acid length. Furthermore, the prediction of the subcellular localization of *GCER* family members was carried out using WoLF PSORT, an online software accessible at https://wolfpsort.hgc.jp/ [105].

### Evolutionary analysis of *CER* in cotton

To investigate the evolutionary link of *CER* between *Arabidopsis thaliana* and the four cotton species (*G. raimondii*, *G. barbadense*, *G. hirsutum* and *G. arboreum*), the alignment of CER protein sequences from these five species was conducted using ClustalW. We created a phylogenetic tree using the MEGA 11 software and the maximum likelihood technique [106], by setting the bootstrap value to 1000, while maintaining default values for all other parameters. The phylogenetic trees were visualized using the iTOL (https://itol.embl.de/) programme.

### Collinearity Analysis and *CER* genes locations on the chromosomes of cotton

The information about the gene location on the chromosomes of *G. raimondii*, *G. arboreum*, *G. barbadense*, and *G*. *hirsutum*, was gained using the cotton reference database. The representation of *CER* gene distribution on the distinct chromosomes of the four cotton species was visually depicted using TBtools [107]. We used the multiple collinearity scan (MCS-scanX) feature in TBtools to assess *CER* genes’ collinearity and using the Basic CIRCOS function in TBtools (v1.108), the collinearity map was constructed.

### Computation of duplicated gene pairs’ selection pressure

Cotton *CER* coding sequences (CDS) were retrieved from the cotton reference database. Following that, the synonymous (Ks) and non-synonymous (Ka) substitution rates were calculated using the TBtools software (v1.108). A Ka/Ks ratio less than one indicates negative selection, a Ka/Ks ratio greater than one shows positive selection, and a Ka/Ks ratio equal to one indicates neutral selection.

### Analysis of motif, domain, and gene structure

The Multiple Em for Motif Elicitation (MEME) available at http://memesuite.org/ was employed to detect conserved motifs within CER proteins, with each motif possessing a p-value lower than 1 *×*10^*−* 5^. TBtools was utilized to examine the *CER* genes’ exon–intron structure. Using the TBtools software a combined image which incorporated phylogenetic trees along with information on domains, motifs, and gene structure, was generated.

### *Cis*-acting element analysis in *CER* promoters

The retrieval of promoter sequences, spanning 2000 base pairs upstream of *CER* genes, and the prediction of *cis*-acting regions were performed using PlantCare (https://bioinformatics.psb.ugent.be/webtools/plantcare/html/). The visualization of the identified elements, in conjunction with the phylogenetic tree, was achieved through the utilization of TBtools.

### Prediction of *GhCER* protein-protein interaction

The STRING 12.0 online tool (available at https://string-db.org/) was used to predict the protein-protein interaction network associated with *GhCER*. which was built on the basis of established *Arabidopsis* proteins. The STRING tool was customized with specific parameters, whereby the comprehensive STRING network was selected as the network type and the required minimum interaction value was set to an average confidence level of 0.4. In addition, the maximum number of interactors displayed was limited to a maximum of 10.

### Prediction of putative miRNAs that target *GhCER* gene and enrichment analyses

The mature sequences of cotton miRNA available from previous works were used to identify the probable miRNA targets in *GhCER* genes [108]. Subsequently, the coding sequences (CDS) of the entire set of 34 *GhCER* genes were provided as input to the psRNATarget server (https://www.zhaolab.org/psRNATarget/) and the parameters for predicting potential miRNA targets in *GhCER* genes were set to default. For the visualization of the interaction network between the *GhCER* target genes and the potential miRNAs, Cytoscape software version 3.10.1 was used (can be downloaded from https://cytoscape.org/download.html). To perform GO and KEGG annotation analyses, all identified *GhCER* gene sequences were submitted to AgriGo v2 and the KEGG database, respectively.

### Analysis of the regulatory network involving plant transcription factors for *GhCER* genes

The prediction of plant transcription factors (TFs) and the subsequent analysis of the regulatory network followed the methodology outlined by Jin et al. (2014), with minor adaptations [109]. Nucleotide sequences including the promoter regions (2,000 base pairs upstream of the *GhCER* genes) were extracted from the cotton genome to predict the upstream transcription factors (TFs) of the *GhCER* genes. These sequences were subsequently deposited in the Plant Transcriptional Regulatory Map (PTRM) at http://plantregmap.gao-lab.org/regulation_prediction.php, with a significance threshold set at *p* ≤ 1e^− 6^ [110]. Cytoscape 3.9.1 software [111] was utilized to create and visualize the regulatory network of transcription factors.

### *CER* gene expression analysis in various tissues and under different stress conditions

The transcriptome data from the Cotton Omics Database (http://cotton.zju.edu.cn/) was utilized to evaluate the gene expression pattern of *CER* genes in different tissue types and under different stress conditions (salt, heat, cold and PEG) [30]. TBtools version 1.108 was employed to generate the heatmap, utilizing FPKM (Fragments Per Kilobase of transcript per Million mapped reads) values.

### Plant materials and treatments

Shayan, an upland cotton variety, tolerant to drought was used for the study. Heat-sterilized sand pots were used to select plump, uniformly sized seeds. Plants were cultivated at 28 °C with 16 hours’ light period, 25 °C with 8 h of dark and a relative humidity of 75%. The plants were divided into experimental and control groups when full development of the third true leaf was observed. The experimental group was subjected to 5% PEG-6000 treatment, while the control group was watered regularly. Leaves and roots collected from both cotton groups were kept at -80 °C for RNA extraction for qRT-PCR assays, 12 h and 24 h after treatment.

### Virus-induced gene silencing of *GhCER04A* in upland cotton

Vectors targeting *GhCER04A* for knockdown using the Tobacco Rattle Virus (TRV)-based virus-induced gene silencing system (VIGS) were constructed [112]. *GhCER04A* and other *CER* genes of G. *hirsutum* always have overlapping regions, so it is important to study the genomic organisation and the overlap between them. We designed specific primers to amplify fragments (avoiding overlapping and conserved regions) to construct VIGS vectors. Non-overlapping regions were identified by genomic positions; non-conserved regions were found using the NCBI Conserved Domain Search Web Service (http://www.ncbi.nlm.nih.gov/Structure/cdd/wrpsb.cgi ). In addition, the specificity of the fragments was verified using BLAST analysis in NCBI [113]. The primer pairs used to construct the vectors are listed in Table S9a. The silenced *GhCER04A* fragment was designed through the SGN VIGS tool (https://vigs.solgenomics.net/) and subsequently inserted into the pTRV2 vector using specific primers. Positive control involved TRV: CLA, while TRV: NULL served as the negative control. The *Agrobacterium tumefaciens* EHA105 strain was utilized for the introduction of the recombinant TRV:*GhCER04A* plasmid. Furthermore, the EHA105 strain was also transformed with the TRV1 (side vector), TRV: NULL, and TRV: CLA plasmids. Cultures of these transformed EHA105 strains were cultivated until the optical density (OD) reached 0.3. Subsequently, the cultures were centrifuged, and the resulting pellets were re-suspended in an infiltration buffer composed of 10 mM MgCl2, 100µM acetosyringone, and 10 mM MES and incubated for 3 h at 25 ± 1 in the dark before infiltrating the cotton seedlings’ cotyledons. Simultaneously the TRV1, TRV: NULL, TRV: CLA, and TRV:*GhCER04A* vectors were also transformed to the competent EHA 105 cells and proceeded for agro-infiltration. The solution was placed for 3 h at room temperature in dark before infiltration into the abaxial side of the cotyledons of cotton seedlings. After one day in the dark, plants were moved to a climate chamber (16-hour light cycle at 12,000 Lx, 25 ℃; 8-hour dark cycle at 0 Lx, 23 ℃). Successful gene silencing was indicated by the albino phenotype in positive control plants. At 4 weeks old, real-time PCR assessed *GhCER04A* expression in TRV: NULL and TRV: *GhCER04A.* Plants were treated with a 20% PEG6000 solution, with ultrapure water as a control. The experimental setup included three biological and three technical replicates, each with five plants.

### Extraction of RNA and qRT-PCR analysis

The hotborate protocol for RNA isolation, was employed to isolate the total RNA from frozen roots [114], which was further converted to cDNA using HiScript III reverse transcription (Qiagen, Germany). The ABI 7500 Fast Real-Time PCR instrument (Thermo, MA, USA) was employed for the qRT-PCR analysis, using the Taq Pro Universal SYBR qPCR Master Mix (Qiagen, Hilden, Germany) with three biological replicates each study. The data was analyzed using the 2^−∆∆Ct^ method, with *GhUBQ7* as the endogenous control. Detailed information about all primers employed in this work can be found in Table S9b.

### Measurement of the physiological parameters associated with drought stress tolerance

In order to elucidate the role of *GhCER04A* in responding to drought stress, plants undergoing different treatments-TRV: NULL, TRV:*GhCER04A*, and mock-were immersed in a 20% PEG6000 solution at the clover leaf stage. This experiment comprised twenty replicates for both drought and well-watered conditions. Subsequent to harvesting leaves from the plants with silencing and the negative control, the levels of malondialdehyde (MDA), water loss rate, and proline levels were assessed, as outlined in previous reports [73]. According to RNA-Seq, most genes showed a sustained increase in response to PEG treatment within 12 h. Therefore, we treated the plant for 12 h, and the real-time and physobiochemical studies were performed on plants treated with 20% PEG6000 for 12 h.

### Electronic supplementary material

Below is the link to the electronic supplementary material.

Supplementary Material 1

Supplementary Material 2

Supplementary Material 3

## Data Availability

Data is provided within the manuscript or supplementary information files.
