# Supplementary material for: Decoding drought resilience: a comprehensive exploration of the cotton Eceriferum (CER) gene family and its role in stress adaptation
Source: BMC Plant Biol. 2024 May 29;24:468. doi: 10.1186/s12870-024-05172-8 (PMC11134665; doi:10.1186/s12870-024-05172-8)
Supplement: Supplementary file 1 — Supplementary Material 1 [file 12870_2024_5172_MOESM1_ESM.docx]

**Figure 1**: Subcellular localisation prediction analysis of the CER gene family in four cotton species. **A**. G*. arboretum*, **B**. G*. raimondii*, **C**. G. *hirsutum*, **D**. G. *barbadense*.
